# Epicardial fat volume is associated with primary coronary slow-flow phenomenon in patients with severe aortic stenosis undergoing transcatheter valve implantation

**DOI:** 10.1186/s12872-024-03927-7

**Published:** 2024-05-16

**Authors:** Maren Weferling, Andreas Rolf, Julia Treiber, Ulrich Fischer-Rasokat, Christoph Liebetrau, Christian W. Hamm, Damini Dey, Won-Keun Kim

**Affiliations:** 1 Department of Cardiology, Kerckhoff Heart and Thorax Center, Benekestr. 2-8, 61231 Bad Nauheim, Germany; 2 German Centre for Cardiovascular Research (DZHK), Partner Site RheinMain, Frankfurt, Germany; 3 Cardioangiological Center Bethanien (CCB), Department of Cardiology, Agaplesion Bethanien Hospital, Frankfurt, Germany; 4 Department of Cardiology, University Hospital of Giessen, Giessen, Germany; 5 Biomedical Imaging Research Institute, Cedars-Sinai Medical Center, 8700 Beverly Blvd, Taper A238, Los Angeles, CA 90048 USA; 6 Kerckhoff Heart and Thorax Center, Department of Cardiac Surgery, Bad Nauheim, Germany

**Keywords:** Epicardial fat tissue, Coronary slow- flow, TIMI frame count, TAVI

## Abstract

**Background:**

Primary coronary slow flow (CSF) is defined as delayed opacification of the distal epicardial vasculature during coronary angiography in the absence of relevant coronary artery stenoses. Microvascular disease is thought to be the underlying cause of this pathology. Epicardial fat tissue (EFT) is an active endocrine organ directly surrounding the coronary arteries that provides pro-inflammatory factors to the adjacent tissue by paracrine and vasocrine mechanisms. The aim of the present study was to investigate a potential association between EFT and primary CSF and whether EFT can predict the presence of primary CSF.

**Methods:**

Between 2016 and 2017, *n* = 88 patients with high-grade aortic stenosis who were planned for transcatheter aortic valve implantation (TAVI) were included in this retrospective study. EFT volume was measured by pre-TAVI computed tomography (CT) using dedicated software. The presence of primary CSF was defined based on the TIMI frame count from the pre-TAVI coronary angiograms.

**Results:**

Thirty-nine of 88 TAVI patients had CSF (44.3%). EFT volume was markedly higher in patients with CSF (142 ml [IQR 107–180] vs. 113 ml [IQR 89–147]; *p* = 0.009) and was strongly associated with the presence of CSF (OR 1.012 [95%CI 1.002–1.021]; *p* = 0.014). After adjustment, EFT volume was still an independent predictor of CSF (OR 1.016 [95%CI 1.004–1.026]; *p* = 0.009).

**Conclusion:**

Primary CSF was independently associated with increased EFT volume. Further studies are needed to validate this finding and elucidate whether a causal relationship exists.

**Supplementary Information:**

The online version contains supplementary material available at 10.1186/s12872-024-03927-7.

## Introduction

Epicardial fat tissue (EFT), located between the myocardium and the visceral border of the pericardium, is the visceral fat depot of the heart. This adipose layer is in immediate contact with the epicardial coronary vessels and the myocardium as it directly surrounds them without the presence of fascial boundaries [1, 2]. Apart from its protective mechanical and thermogenic properties [3], EFT is a highly metabolically active endocrine organ with both anti-inflammatory and pro-inflammatory as well as pro-atherogenic properties: it produces and releases cytokines such as interleukin (IL)-1, IL-6, tumor necrosis factor (TNF)-α, and other bioactive molecules [4]. Due to the proximity of EFT to the epicardial vessels and the myocardium, these agents are released directly into these adjacent structures by vasocrine or paracrine mechanisms [3].

Primary coronary slow flow (CSF) is defined as delayed distal opacification of the coronary epicardial vasculature in the absence of a relevant epicardial stenosis. In theory, reduced contrast flow mimics decreased coronary blood flow and subsequently mirrors increased elevated microvascular resistance and thus coronary microvascular dysfunction (CMD) [5]. The Coronary Vasomotion Disorders International Study (COVADIS) group recently published standardized criteria for diagnosing microvascular angina, and the presence of the CSF phenomenon is named as one criterion for evidence of CMD [6]. However, just recently, the association between CSF and CMD has been questioned by two studies showing no relationship between primary CSF and coronary flow reserve (CFR) or the index of microcirculatory resistance (IMR), two established parameters of CMD [7, 8].

Vast amounts of data indicate a strong relationship between the magnitude of coronary artery disease (CAD), coronary plaque burden, and coronary calcification and the amount of EFT [9, 10]. Data on a potential association between primary CSF and EFT, however, are scarce. Our study group recently found that patients with primary CSF had a higher EFT thickness, measured via transthoracic echocardiography (TTE), than matched controls without CSF [11]. The aims of the present study were to investigate the relationship of EFT volume, measured by computed tomography (CT), and the presence of primary CSF in patients undergoing transcatheter valve implantation (TAVI) and, further, to evaluate the potential of CT-derived EFT volume to predict CSF and compare that with EFT thickness measured via TTE.

## Methods

### Study population

The original study cohort was previously described [12]. In brief, between January 2016 and August 2017, *n* = 560 consecutive patients with severe degenerative aortic stenosis planned for TAVI were included in the analysis. EFT volume was determined retrospectively from CT data using dedicated software (see below). For the present analysis, patients with relevant CAD (defined as an epicardial stenosis ≥ 50% and/or a history of percutaneous coronary intervention (PCI) and/or myocardial infarction) (*n* = 346) or impaired left ventricular systolic function (ejection fraction < 50%) (*n* = 25) were excluded. In addition, patients without coronary angiograms or only angiograms of insufficient quality for detection of the TIMI frame count (TFC) or without information of the frame rate used (*n* = 101) were excluded. The final study cohort comprised 88 patients. The study was conducted in adherence to the Declaration of Helsinki. The study was approved by the Ethics Committee of the Justus-Liebig University of Giessen, Giessen, Germany, and the need for patients’ informed consent was waived due to the retrospective design of the study.

### Multidetector computed tomography (MDCT) analysis before TAVI

Electrocardiogram-gated MDCT examinations were performed with a 128-slice or a 384-slice dual-source scanner (SOMATOM® Definition or Force; Siemens Healthineers, Forchheim, Germany) as previously described [13]. Reconstructions were carried out using a cardiac-gated B26f or I26f algorithm with a slice thickness of 0.6 mm in systole at 35% and in diastole at 70% of the cardiac cycle [12].

### Measurement of EFT volume

**Fig. 1:**
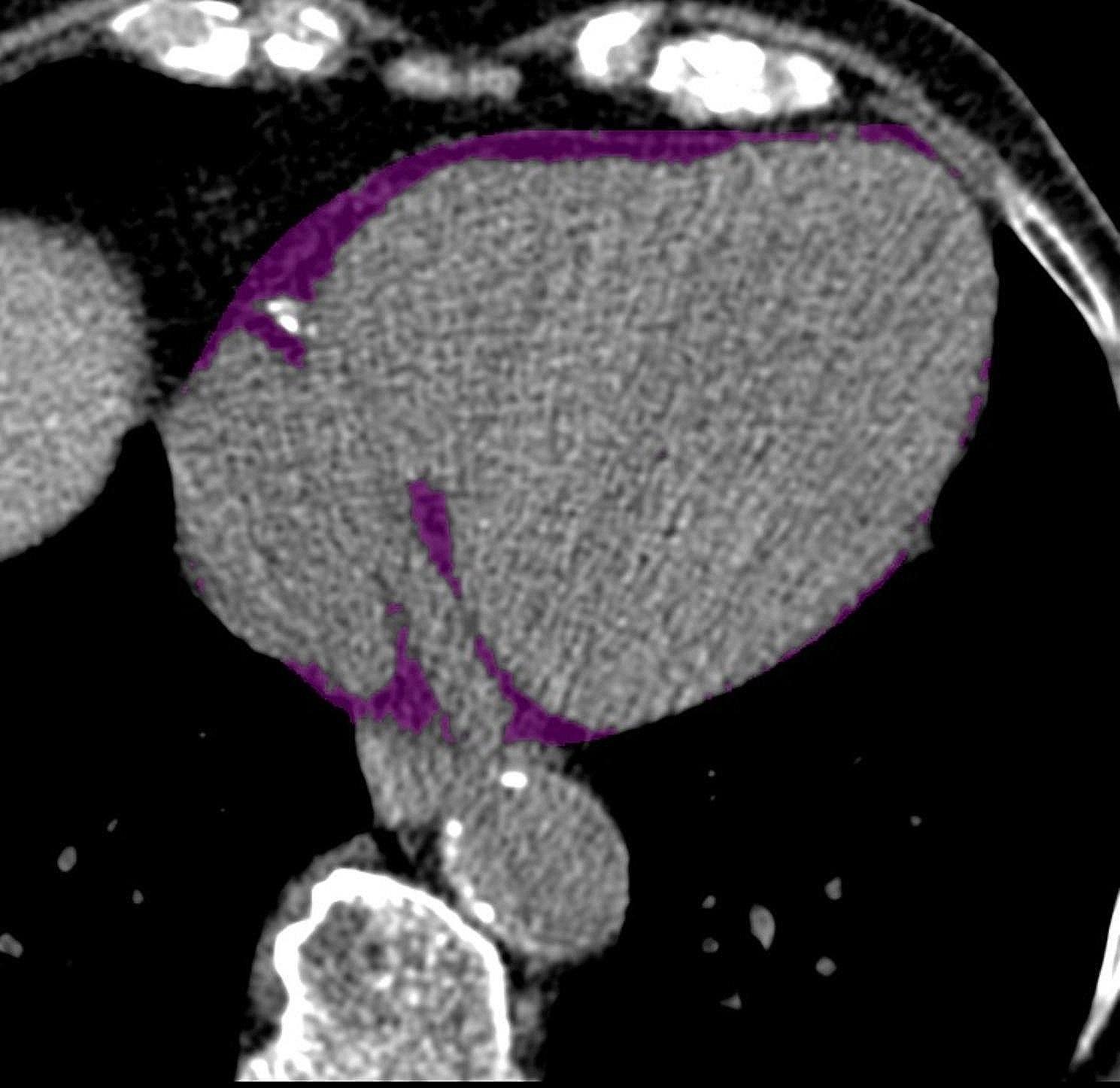
Example of EFT volume measurement. CT-derived slice of the heart in the axial view with tracing of the pericardial border. The areas marked in purple represent EFT. Abbreviations: EFT = epicardial fat tissue; CT = computed tomography

Quantification of EFT volume was accomplished using an established fully automated deep-learning algorithm which is a module of research software (QFAT V2.0, Cedars-Sinai Medical Center, Los Angeles, CA, USA) that has been validated and tested in a large multicenter study [14]. For EFT volume measurements, the bifurcation of the pulmonary trunk was used as the upper boundary, and the most inferior slice with any portion of the heart was used as the lower limit, as recommended by Dey et al. [15]. In cases where the automatically generated tracings of the pericardial border were inadequate due to insufficient image quality, the tracings were adjusted manually. EFT volume was calculated in ml. An example of EFT volume measurement is shown in Fig. 1.

### Measurement of the TFC

The TFC was determined according to Gibson et al. [16]: on the basis of a frame rate of 30 frames/sec, impaired coronary flow was defined as > 21.0 ±3 standard deviation (SD) frames that are needed for the contrast dye to reach certain distal anatomic landmarks in the epicardial vessels. CSF was diagnosed when two SDs were exceeded (> 27 frames/sec) in at least one epicardial coronary vessel. For the left anterior descending coronary artery (LAD), the so-called “whale’s tail” is determined as the distal landmark, meaning the bifurcation of the vessel at the apex; for the circumflex artery (RCX) the distal landmark is the most distal bifurcation of the last marginal branch; for the right coronary artery (RCA) it is the first side branch of the posterolateral branch [16]. The frame count starts when three criteria are met: the dye must fill or nearly fill the entire width of the origin of the vessel, touch both vessel borders, and have forward flow. Due to the vessel’s greater length, the TFC of the LAD is divided by 1.7 [16].

### Measurement of EFT thickness on echocardiography

EFT thickness was measured according to the work of Iacobellis et al. [17]. In brief, in the parasternal long-axis view (PLAX) of two-dimensional echocardiographic imaging, EFT thickness was measured in mm perpendicular to the right ventricular free wall with the aortic annulus as landmark. To avoid false measurements, careful attention was paid to the discrimination between epicardial and pericardial fat layers. Whenever possible, measurements were made for a total of 3 cardiac cycles during end-systole and the mean value was calculated [11].

### Statistical methods

Continuous data are presented as mean with standard deviations (± SD) or as median with interquartile range (IQR), as appropriate. Metric parameters were tested for normal distribution pattern via the Shapiro-Wilk test. Two groups were created according to the presence of primary CSF (“CSF” and “non-CSF”). Comparison of continuous variables between the groups was performed with the Mann-Whitney-U test if not normally distributed and with an unpaired t-test if normally distributed. Categorical parameters were compared via the Chi-squared test. Univariate and multivariable logistic regression analysis (after adjustment for age and sex) was performed to test whether EFT volume is an independent predictor of CSF. The association between CT-derived EFT volume and TTE-derived EFT thickness was assessed via Pearson correlation analysis. Receiver operator characteristics (ROC) curve analysis was used to evaluate EFT volume and EFT thickness in terms of the ability to predict primary CSF, and the results were compared using the DeLong’s test. Significance was assumed when a two-sided p-value ≤0.05 was determined, indicating that the null hypothesis was rejected. SPSS Version 22.0 (IBM, Armonk, New York, USA) was used for all statistical analyses except for the DeLong’s test, which was applied using STATA17 (StataCorp, College Station, TX, USA).

## Results

### Patient characteristics

**Fig. 2:**
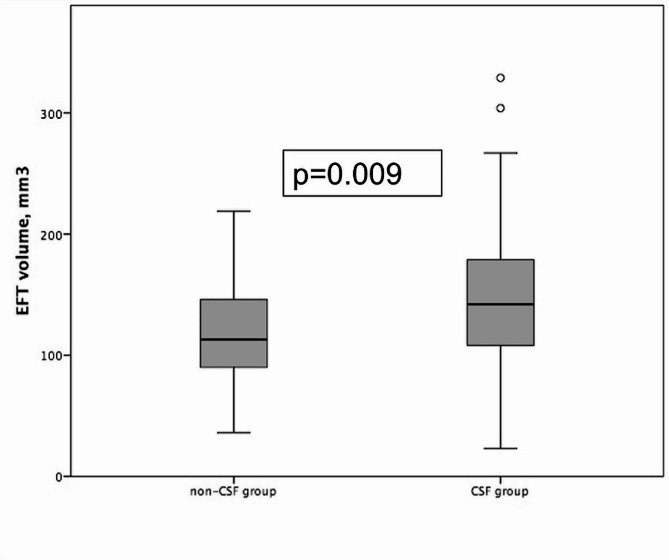
Comparison of EFT volumes between the non-CSF and the CSF cohorts Box-and-whiskers plot showing that in patients with CSF, EFT volume is markedly higher than that of non-CSF patients. Abbreviations: EFT = epicardial fat tissue

A total of 88 patients (77.3% female) were included in the analysis. The median age was 81 years [IQR 78–85]. The median EFT volume was 122 ml [IQR 94–155]. CSF was observed in 39 (44.3%) patients. EFT volume was higher in the CSF group than in the non-CSF group (142 ml [IQR 107–180] vs. 113 ml [IQR 89–147]; *p* = 0.009) (Fig. 2). No relevant differences were observed for age and BMI between the groups; however, more patients in the CSF group were male (33.3% vs. 14.3%; *p* = 0.034). A history of cardiac decompensation prior to TAVI was less frequent in the group with CSF (32.7%vs. 12.8%; *p* = 0.03), and these patients had higher baseline mean transvalvular gradients (52 mmHg [IQR 42–61] vs. 41 mmHg [IQR 30–58]; *p* = 0.023). No differences were observed for baseline ECG characteristics, serum creatinine/estimated glomerular filtration rate (eGFR), C-reactive protein (CRP), or leucocyte counts between the groups. Table 1 shows the baseline characteristics of the cohort overall and both subgroups.

### Coronary vessel involvement in CSF patients

One-vessel involvement (i.e., only one coronary vessel was affected by slow flow) was most common in CSF patients (51.3%), and 2- and 3-vessel involvement was present in 30.8% and in 17.9% in the CSF group, respectively. The circumflex artery was most frequently affected (66.7% of CSF cases), the RCA exhibited slow flow in 53.8%, and the LAD was affected in 46.2%. There was no difference in EFT volume between CSF patients with 1- versus patients with 2- or 3-vessel involvement (130 ml [IQR 105–188] vs. 142 ml [IQR 120–167]; *p* = 0.989).

### Epicardial fat volume and prediction of CSF

**Fig. 3:**
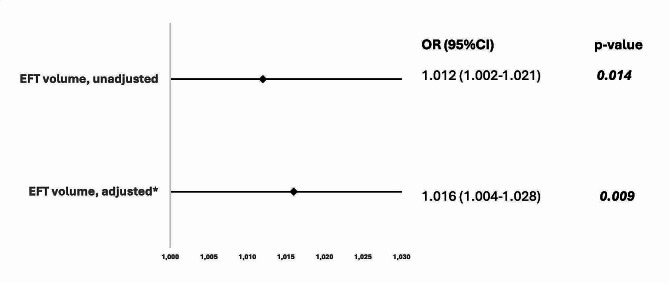
Unadjusted and adjusted predictive value of EFT volume for CSF. After adjustment* for sex, mean aortic gradient, and prior cardiac decompensation, CT-derived EFT volume still predicts CSF. Abbreviations: EFT = epicardial fat volume; CSF = coronary slow flow

In univariate logistic regression analysis, EFT volume strongly predicted the presence of primary CSF (OR 1.012 [95%CI 1.002–1.021]; *p* = 0.014). After adjustment for sex, mean aortic gradient, and prior cardiac decompensation, EFT volume was still an independent predictor of CSF (OR 1.016 [95%CI 1.004–1.028]; *p* = 0.009). Figure 3 shows Forest plots of the unadjusted and adjusted predictive value of EFT volume. In contrast, EFT thickness was tested in the same regression model and was found not to be an independent predictor of CSF (OR 1.139 [95%CI 0.884–1.466]; *p* = 0.314)

### EFT volume versus thickness in predicting CSF

TTE-derived measurement of EFT thickness was possible in 85 of the 88 patients. In the remaining 3 patients image quality was not sufficient for a proper evaluation of EFT in PLAX. The median EFT thickness was 4.2 mm [IQR 3.4–5.3] for the overall cohort. CSF patients had a numerically higher EFT thickness than non-CSF patients (4.7 mm [IQR 3.6–5.6] vs. 3.8 mm [IQR 3.2–5.2]; *p* = 0.118), although this difference was not significant.

**Fig. 4:**
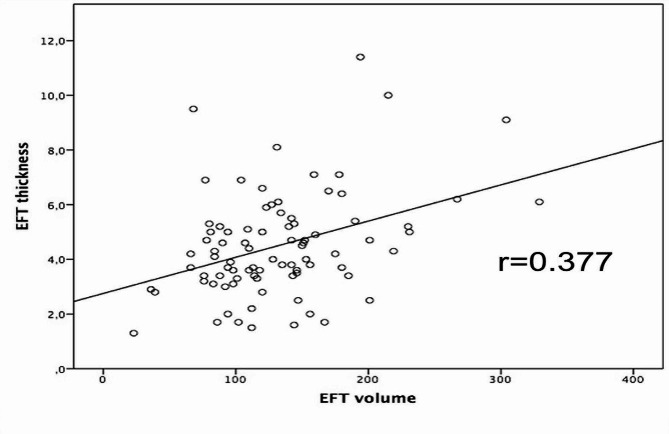
Correlation analysis of TTE-derived EFT thickness and CT-derived EFT volume. EFT thickness and EFT volume correlate moderately (*r* = 0.377; *p* < 0.001). Abbreviations: EFT = epicardial fat tissue

EFT volume and thickness showed moderate correlation (*r* = 0.377; *p* < 0.001) (Fig. 4). In ROC curve analysis, EFT volume predicted CSF with a c-statistic value of 0.645 [95%CI 0.53–0.76]; *p* = 0.022. EFT thickness predicted CSF with an AUC of 0.599 [95%CI 0.48–0.72]; *p* = 0.118) (*Suppl. Figure 1*). However, when the two ROC curves were compared with each other, no significant difference was present (*p* = 0.54).

## Discussion

The present analysis showed that in TAVI patients without relevant CAD (i.e., no stenosis ≥ 50% in an epicardial vessel) and normal or near-normal left ventricular systolic function (i.e., LVEF ≥ 50%) (1) EFT volume was higher in patients with CSF than in those without CSF; (2) EFT volume was an independent predictor of CSF, whereas EFT thickness was not (3) EFT volume and EFT thickness were moderately correlated.

While a vast amount of data exists regarding the relationship between the amount of EFT and the presence of CAD, coronary calcification, and plaque vulnerability [18–20], little is known about a potential association between EFT and primary CSF. In fact, our study group was the first and, to our knowledge, thus far the only one investigating the role of TTE-derived EFT thickness in patients undergoing coronary angiography with suspected CAD and potentially concomitant CSF [11]. The main finding was that in 48 patients EFT thickness was associated with the presence of CSF. The rationale for this investigation was the hypothesis that microvascular and endothelial dysfunction are the underlying mechanism of primary CSF: since the coronary microvasculature, in contrast to the epicardial vessels, constitutes the main compartment of resistance and thus regulation of blood flow, anatomic and/or functional pathologies such as morphological alterations or endothelial dysfunction of the small vessels could have a substantial impact on blood flow and thus affect contrast dye filling. This idea is underlined by histological studies showing “small-vessel disease” in myocardial biopsies from patients with CSF [21, 22]. From a clinical point of view, this hypothesis was further affirmed by Fineschi et al. who invasively investigated the resting index of microvascular resistance (IMR) and coronary flow reserve (CFR) in 15 patients (8 with CSF and 7 controls) and found that IMR was significantly increased in CSF patients [23]. From a pathophysiological point of view, EFT, with its close anatomical proximity to the coronary vessel wall [1–3], is able to release proinflammatory cytokines such as IL-1, IL-6, IL-8, and TNF-α that directly diffuse into the vessel wall by paracrine mechanisms [24, 25], and these cytokines could thus play an important role in CSF by inducing morphological and functional alterations in the coronary microvasculature. To the best of our knowledge, this association has not been investigated thus far. Our findings support the hypothesis that there is an association between EFT and primary CSF, and further studies are warranted to validate our results and to elucidate the pathophysiological relation between EFT and CSF.

In the past, many investigators suggested that CSF is a surrogate for microvascular dysfunction based on the assumption that from a pathophysiological point of view elevated microvascular resistance must have a negative impact on coronary flow. However, clear evidence for this theory does not exist, and there are studies showing no association between parameters of diastolic dysfunction that could be a surrogate for microvascular disease and CSF [26]. Our study did not investigate the presence of microvascular dysfunction or diastolic dysfunction; however, it must be assumed that patients with severe aortic stenosis are very prone to both entities due to increased myocardial wall thickness and subsequent impaired relaxation of the left ventricle, elevated left ventricular end-diastolic pressure, and thus increased pressure on the microvasculature [27–29]. The fact that we detected a difference in EFT volume between patients with and without CSF in a study cohort with a high likelihood of microvascular dysfunction and diastolic dysfunction implies that CSF might not be a surrogate for these entities and that other factors, such as a potentially increased inflammatory environment due to higher amounts of EFT, play a role in the development of CSF. This hypothesis is underlined by two studies that were just recently published. Harkin et al. investigated 104 patients for the presence of CSF (defined as opacification of the distal vasculature in more than three heartbeats) and either abnormal CFR or IMR or both [7]). Patients who were diagnosed with CSF had similar CFR and IMR values compared with patients with normal coronary flow: 17.4% vs. 25.9% (*p* = 0.40) for abnormal CFR and 43.5% vs. 32.1% (*p* = 0.31) for abnormal IMR [7]. In a larger patient cohort comprising 508 patients (48% with CSF), CMD (defined as either CFR < 2.5 or IMR ≥ 25) was also not more prevalent in patients with CSF than in those without [8].

In contrast to the findings of our previous study [11], TTE-derived EFT thickness was not able to predict CSF in the present analysis. This discrepancy may be due to distinct differences between the two study cohorts. In our previous study, the cohort comprised patients with angiographically obvious primary CSF who were retrospectively selected based on their coronary angiogram records containing the terms “coronary slow flow”, “delayed opacification”, and “delayed flow” [11]. As the interventionalists used these terms only when the slow flow was obvious, there was most likely a selection bias towards very severe forms of CSF. Conversely, in the present study, the patients were extracted out of a previously described study cohort in which CT-derived EFT volume was originally investigated with regard to the development of conduction disturbances in TAVI patients [12]. These patients’ coronary angiograms were investigated in the present study for the presence of CSF according to the definition by Gibson et al. [16]. Thus, it is conceivable that TTE-derived EFT thickness, as measured in two dimensions which is naturally less precise than measurement of CT-derived EFT volume, is nonetheless able to predict CSF when this phenomenon is present in its more pronounced form.

In the present study, EFT volume and EFT thickness were correlated moderately with each other (*r* = 0.377, *p* < 0.001). This finding is in line with the results of Kim et al. in the CAESAR study: in over 2,200 patients, CT-derived EFT volume and TTE-derived EFT thickness were compared in terms of their association with the coronary artery calcium score [19]. The authors found a significant association of the absolute values of both EFT volume and EFT thickness with coronary calcification. The two parameters showed a moderate correlation with each other (*r* = 0.374; *p* < 0.001), which is similar to the correlation in our study [19].

Interestingly, in contrast to the lack of data available on primary slow flow and EFT, there are some data on the association between the amount of epicardial fat and the no-reflow phenomenon in the acute setting of myocardial infarction. Zencirci et al. found that in 114 patients presenting with ST-segment elevation myocardial infarction (STEMI), impaired ST-segment resolution (defined as < 70%), as a surrogate parameter for no-reflow, was more common in patients with greater EFT thickness than in patients having adequate ST segment resolution [30]. Furthermore, EFT thickness independently predicted the presence of the no-reflow phenomenon (OR 1.43 [95%CI 1.13–1.82]; *p* = 0.003). The study authors postulate that based on the pro-inflammatory properties, EFT influences the coronary microvasculature and potentially makes it more prone to no-reflow in the acute setting. However, the authors correctly state that it is not clear whether EFT plays an active role in the setting of no-reflow or whether it is only a bystander [30]. A similar result was found by Mohamed, who analyzed the presence of increased EFT thickness (defined as > 5 mm measured in PLAX in TTE) in comparison with the no-reflow phenomenon in 113 patients presenting with acute STEMI [31]. Here, EFT thickness also significantly correlated with the presence of the no-reflow phenomenon, with 89.9% of patients having an EFT thickness > 5 mm and showing TIMI flow < III. Although the “acute” patient subset of these two studies obviously differs from our cohort and cannot be simply extrapolated to the “chronic” setting of primary CSF, these results strengthen our hypothesis that EFT plays a major role in CSF. It is of utmost importance that more studies are conducted to further investigate this issue.

### Limitations

Several limitations of our study should be addressed. First, as this is a single-center investigation, our findings may not be generalizable to other patient groups or to the population overall. Second, the study cohort is rather small, weakening the statistical power of our analysis. It remains speculative whether EFT thickness would also have been able to predict CSF with a larger sample size. Although the original study cohort comprised over 500 patients, a large proportion of patients had coronary angiograms that did not fulfill the criteria defined by Gibson et al. for assessment of TFC. This was not due to insufficient quality but rather to the fact that filming of the coronary system was started when the coronary tree was completely filled with contrast dye (in order to reduce radiation) and thus TFC assessment was impossible. Also, the exclusion of CAD patients, which was necessary as the presence of CAD can have an impact on coronary flow, led to a further reduction of the original cohort. Due to this exclusion criterion, as CAD is more prevalent in male than in female patients, our final study cohort had a female predominance. It cannot be excluded that this sex-specific bias had an influence on the results. Third, our study is hypothesis generating; thus, although an association between EFT and CSF was found, a potential pathophysiological relationship needs to be verified. Prognostic implications potentially associated with CSF were beyond the scope of this study and should be addressed in large-scale studies. Fourth, the definition of coronary slow flow by TFC according to Gibson et al. was originally set up for patients with acute myocardial infarction after thrombolytic therapy in the mid-1990s [16] and not primarily for patients with coronary slow flow and normal epicardial vessels. However, leading researchers in the field of primary CSF used this definition for the diagnosis of primary CSF [5]. Apart from the alternative estimation of CSF by using the TIMI-flow grading method, which is more subjective as it depends on visual assessment of the opacification of the coronary vessel, there does not appear to be any other way to define primary CSF. Lastly, the study population is a specific one comprising patients suffering from severe aortic stenosis. The impact of this disease itself on coronary flow is unclear, and therefore our findings cannot be extrapolated to a “healthier” population with primary CSF. The reason for using this patient population was a practical one: almost all TAVI patients undergo CT scanning and coronary angiography for procedure planning. Conversely, patients suspected to have CAD who primarily receive a CT scan with the result of having normal or near-normal coronary arteries usually do not proceed to coronary angiography, as CAD is excluded. Therefore, this kind of patient population was not suitable for our analysis. However, the main aim of this study was to investigate whether an association between EFT and CSF exists at all, and if our positive finding can be confirmed by other investigators, the second step would be to look for differences in different patient populations.

## Conclusion

Increased CT-derived EFT volume was significantly associated with the presence of primary CSF in patients undergoing TAVI. Further studies with larger sample sizes are needed to validate these findings and to elucidate the pathophysiological role of EFT in patients with primary CSF.

**Table 1:** Baseline characteristics of the total cohort and CSF- and non-CSF groups

	Total cohort (*n* = 88)	CSF (*n* = 39)	Non-CSF (*n* = 49)	*p*-value
*General characteristics*				
Age, years	81 [75–85]	81 [79–85]	81 [77–85]	0.765
Sex, female	68 (77.3)	26 (66.7)	42 (85.7)	***0.034***
Body mass index, kg/m^2^	27.6 (SD 4.1)	29.7 (SD 3.7)	26.2 (SD 3.8)	0.071
Hypertension	78 (88.6)	33 (84.6)	45 (91.8)	0.289
Diabetes mellitus	18 (20.5)	7 (17.9)	11 (22.4)	0.603
Hyperlipidemia	22 (25)	8 (20.5)	14 (28.6)	0.386
Prior stroke	11 (12.5)	5 (12.8)	6 (12.2)	0.935
COPD	22 (25)	11 (28.2)	11 (22.4)	0.536
Prior decompensation	21 (23.9)	5 (12.8)	16 (32.7)	***0.03***
Syncope	18 (20.5)	6 (15.4)	12 (24.5)	0.293
NYHA class > 2	73 (83)	33 (84.6)	40 (81.6)	0.712
*ECG parameters*				
AV block I	12 (13.6)	7 (17.9)	5 (10.2)	0.293
Left bundle branch block	6 (6.8)	1 (2.6)	5 (10.2)	0.158
Right bundle branch block	11 (12.5)	6 (15.4)	5 (10.2)	0.465
*Echocardiographic parameters*				
Ejection fraction, %	65 [65–65]	65 [65–67]	65 [65–65]	0.8
Mean transvalvular gradient, mmHg	45 [36–59]	52 [42–61]	41 [30–58]	***0.023***
Aortic valve area, cm^2^	0.7 [0.5–0.8]	0.6 [0.5–0.8]	0.7 [0.5–0.8]	0.621
*Blood parameters*				
Leucocytes, /nl	7.3 [6.6–8.9]	7.3 [6.6–8.9]	7.2 [6.5–8.6]	0.655
Creatinine, mg/dl	0.9 [0.7–1.1]	0.9 [0.7–1.1]	0.9 [0.7–1.2]	0.966
eGFR, ml/min/1.73 m^2^	73 [56–90]	73 [57–90]	73 [51–87]	0.718
CRP, g/dl	0.3 [0.1–0.8]	0.3 [0.1–0.7]	0.3 [0.2-1]	0.238

Values represent number (%), mean (SD), or median [IQR]. Significant p-values (< 0.05) are depicted in bold and italics

Abbreviations: CSF = coronary slow flow; SD = standard deviation; COPD = chronic obstructive pulmonary disease; NYHA = New York Heart Association; ECG = electrocardiographic; AV block = atrio-ventricular block; eGFR = estimated glomerular filtration rate; CRP = C-reactive protein

### Electronic supplementary material

Below is the link to the electronic supplementary material.

Supplementary Material 1

## Data Availability

The datasets generated and/or analysed during the current study are not publicly available due to ethical or legal reasons, e.g., public availability would compromise patient confidentiality or participant privacy, but are available from the corresponding author on reasonable request.

## Abbreviations

CAD: Coronary artery disease
CFR: Coronary flow reserve
CMD: Coronary microvascular dysfunction
CSF: Coronary slow flow
EFT: Epicardial fat tissue
IMR: Index of microvascular resistance
IQR: Interquartile range
LAD: Left anterior descending
MDCT: Multidetector computed tomography
PLAX: Parasternal long-axis view
PCI: Percutaneous coronary intervention
RCA: Right coronary artery
RCX: Circumflex coronary artery
STEMI: ST-segment elevation myocardial infarction
TAVI: Transcatheter aortic valve implantation
TFC: TIMI frame count
TTE: Transthoracic echocardiography
