# Supplementary material for: Epicardial fat volume is associated with primary coronary slow-flow phenomenon in patients with severe aortic stenosis undergoing transcatheter valve implantation
Source: BMC Cardiovasc Disord. 2024 May 16;24:253. doi: 10.1186/s12872-024-03927-7 (PMC11097472; doi:10.1186/s12872-024-03927-7)
Supplement: Supplementary file 1 — Supplementary Material 1 [file 12872_2024_3927_MOESM1_ESM.docx]

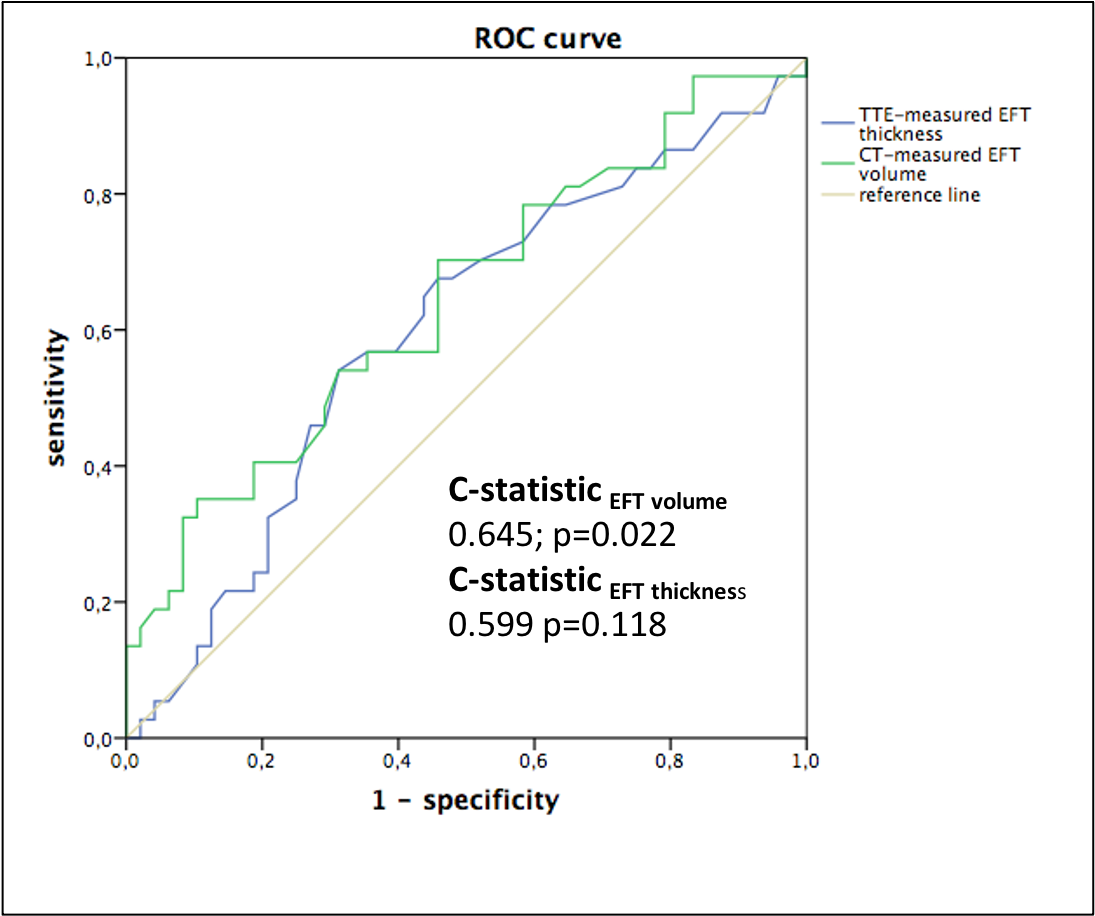


**Title**

ROC curve analysis of TTE-derived EFT thickness and CT-derived EFT volume in terms of the presence of CSF

**Legend**

In contrast to EFT thickness, CT-derived EFT volume moderately predicts CSF (c-statistic 0.645; p=0.022).
